# Admission Hyperglycemia as a Predictor of COVID-19 Pneumonia, Cytokine Release Syndrome Progression, and Clinical Outcomes in a Tertiary Care Hospital

**DOI:** 10.7759/cureus.27021

**Published:** 2022-07-19

**Authors:** Sajjad Ali, Omar S Khan, Ayman m Khalil, Ahmad K Odeh

**Affiliations:** 1 Infection Control Department, Sultan Bin Abdulaziz Humanitarian City, Riyadh, SAU; 2 Medical Affairs Department, Sultan Bin Abdulaziz Humanitarian City, Riyadh, SAU

**Keywords:** clinical outcomes, pneumonia, cytokine release syndrome, admission hyperglycemia, covid-19 infection

## Abstract

Introduction

Diabetes and coronavirus disease 2019 (COVID-19) are interrelated. The presence of hyperglycemia per se during COVID-19 infection regardless of diabetes status has been associated with poor prognosis and increased risk of mortality.

Objectives

The main aim of the current study was to assess the association between admission hyperglycemia and COVID-19 outcomes.

Methods

This is a retrospective cohort study including 315 patients, mainly employed in the facility, who presented to the emergency department or were admitted with confirmed COVID-19 infection from April 2020 to August 2021.

Results

The mean age of the studied cohort was 40.2±12.5 years, where 59.68% were males and 37.7% were symptomatic. Older age, male gender, history of diabetes and hypertension, and elevated C-reactive protein (CRP) and lactate dehydrogenase (LDH) levels were associated with a significantly increased risk of developing cytokine release syndrome (CRS). Admission hyperglycemia was significantly associated with poor outcomes. The time to negativity was 9.30±0.1 days for asymptomatic patients; however, it increased significantly according to clinical presentation, presence of comorbidities, and severe outcomes, in patients with cytokine release syndrome.

Conclusions

Admission hyperglycemia was associated with an increased risk of progression to critical condition in patients hospitalized with COVID-19 independent of the history of diabetes. Therefore, it should not be overlooked but instead should be detected and appropriately treated to improve outcomes. In addition, post-COVID-19 care should be individualized, where severe cases require almost double the time needed by mild cases to convert to negative.

## Introduction

The coronavirus disease 2019 (COVID-19) pandemic has undoubtedly become a global disaster in 2020 [1]. In addition to its known role in the respiratory system, severe acute respiratory syndrome coronavirus 2 (SARS-CoV-2) has been shown to influence the endocrine system, particularly the pancreas [2,3]. Diabetes and COVID-19 have an interrelated relationship, where, on one hand, diabetes is associated with an increased risk of severe COVID-19 infection. On the other hand, new-onset diabetes, and severe metabolic complications of preexisting diabetes, have been observed in patients with COVID-19 [4,5]. There are several mechanisms proposed for such a relation. Firstly, human pancreatic alpha and beta cells have been demonstrated to be susceptible to SARS-CoV-2 infection [6]. At the same time, SARS-CoV-2 has been shown to infect and replicate in human endocrine and exocrine pancreatic cells [7]. Secondly, COVID-19 infection, through direct B-cell tropism, induces an inflammatory process that would interfere with normal B-cell function [8].

Medical literature had collected enough data to show that patients with diabetes suffering from COVID-19 infection would manifest poor outcomes that are related to hyperglycemia, as shown in a meta-analysis by Lee et al. [9]. The presence of hyperglycemia per se during COVID-19 infection in patients with diabetes or patients without diabetes is associated with poor prognosis and increased mortality risk [10]. This could be explained by the cytokine storm syndrome that results from increased inflammatory cytokines related to insulin resistance and consequent hyperglycemia [2]. Such excessive production of pro-inflammatory cytokines leads to acute respiratory distress syndrome (ARDS) aggravation and widespread tissue damage, resulting in multi-organ failure and death [11]. Therefore, early intervention to avoid or treat cytokine storm syndrome should be considered in such cases to improve outcomes and decrease mortality [12].

To better understand this phenomenon, this study being in a closed medical community that provides early diagnosis and intervention with low mortality and had extensive clinical and biochemical evaluation would give a better understanding to correlate hyperglycemia and COVID-19 infection in relation to their outcomes.

## Materials and methods

Methods

Study Design and Participants

This is a retrospective cohort study that was conducted in Sultan Bin Abdulaziz Humanitarian City (SBAHC), a 512-bed capacity tertiary care hospital with around 2,000 employees. This study included adult patients, mainly city staff, who presented to the emergency department or were admitted with a confirmed COVID-19 infection from April 2020 to August 2021. To avoid potential confounding effects, patients with malignancy, human immune deficiency virus, and immune deficiency syndromes were excluded due to the expected worst outcomes in such populations. A total of 315 patients were included in the study.

Definition

Case Definition

The case definition for this study was based on laboratory confirmation through nasal/pharyngeal swab specimens. Confirmed cases tested positive for SARS-CoV-2 nucleic acid using a real-time reverse transcription-polymerase chain reaction (RT-PCR) assay.

Hyperglycemia

Hyperglycemia was defined as having a random blood glucose (BG) level greater than 140 mg/dL at presentation regardless of the preexistence of diabetes based on American Diabetes Association guidelines [13].

Severity Categories

Severity categories included mild (mild respiratory symptoms and normal lung examination), severe (pneumonia, fever, cough, hypoxemia, or respiratory distress), and critical (above symptoms and cytokine release syndrome, acute respiratory distress syndrome, shock, other life-threatening organ dysfunction, or death).

Time to Negativity

The time length to negativization was defined as the period between the beginning of symptom onset confirmed by the first RT-PCR-positive result to the day of the second successive negative ribonucleic acid (RNA) SARS-CoV-2 test result, as proposed in World Health Organization guidelines [14].

Data collection

Data were collected from electronic medical records using a predesigned case report form through a trained research physician. Data on patients’ demographics including age, gender, nationality, height, and weight; past clinical history, including a history of potential complications such as diabetes mellitus, hypertension, and hyperlipidemia; and clinical presentation suggestive of COVID-19 in the form of flu-like symptoms, fever, cough, shortness of breath, loss of smell or taste, headache, abdominal pain, and abnormal bowel motion, were collected. Each patient was subjected to extensive laboratory investigations that included blood glucose, WBC count, lymphocyte count, lactate dehydrogenase (LDH), C-reactive protein (CRP), D-dimer, troponin, and liver function; management modalities; and outcomes including complications, discharge, intensive care unit (ICU) admission, and mortality. All symptomatic patients were subjected to chest X-rays with or without chest computed tomography (CT) based on their radiological findings.

Severe and critical patients were hospitalized, and the hospital course was documented, including the daily clinical assessment that reported complications and outcomes in addition to therapeutic intervention. Patients were closely monitored until full recovery or ICU admission or mortality.

Statistical analysis

Descriptive statistics were conducted, where categorical variables were presented as counts and proportions, while continuous variables were presented as means and standard deviations (SD). The chi-square test (χ2 ) was used for categorical data, while an unpaired t-test was used for numerical data. Logistic regression was performed to evaluate the association between admission hyperglycemia and cytokine release syndrome (CRS) and pneumonia. Time to testing negative was investigated using survival analysis by a Kaplan-Meier plot and compared using the log-rank test. Data analysis was conducted using the Statistical Package for Social Sciences (SPSS) version 21.0 (IBM Corp., Armonk, NY, USA), and a p-value of <0.05 was considered statistically significant.

The study was approved by the institutional review board in Sultan Bin Abdulaziz Humanitarian City (IRB review number 38-2020-IRB). Written informed consent was waived owing to the rapid emergence of this infectious disease and retrospective study design. The study used anonymous clinical data for analysis. The study was performed in accordance with the ethical standards laid down in the 1964 Declaration of Helsinki.

## Results

In this study that has investigated cases of COVID-19 infection in a closed community of a medical rehabilitation institute, 315 patients have suffered from this infection from April 2020 to August 2021. These patients were mainly employees and had a mean age of 40.2±12.5 years, where 59.68% were males. The mean BMI was 25.4±4.2 kg/m^2^, and it was significantly higher among females. Hypertension followed by hyperlipidemia and diabetes were the most frequent comorbidities at a rate of 12.69%, 10.79%, and 8.88% respectively.

During hospitalization for COVID-19 infection, 12.38% had developed pneumonia, where the majority were males. CRS occurred in 5.88% of the patients, and it was more predominant among males. Single organ failure was observed in 12 (3.8%) patients, while multi-organ failure was reported in eight (2.53%) patients. Direct exposure to patients was reported in 21.26% of the cases, especially females (Table 1).

**Table 1:** General characteristics of COVID-19 patients. *Newly diagnosed DM cases + known cases, **organ failure during hospitalization

Parameters	All (N=315) (100%)	Males** (n=188) (59.68%)	Females** (n=127) (40.31%)	p-value
Age (years) (mean±SD)	40.2±11.50	41.7±11.50	38.1±11.40	0.0066
BMI (kg/m^2^) (mean±SD)	25.4±4.20	25.2±4.1	26.4±4.20	0.018
Overweight (n (%))	101 (32.06)	63 (33.51)	38 (29.92)	0.503
Obese (n (%))	49 (15.55)	24 (12.76)	25 (19.68)	0.092
Nationality (n (%))				
Saudi	104 (33)	48 (25.50)	55 (43.40)	0.0010
Non-Saudi	213 (67)	140 (74.50)	72 (56.60)	0.0009
Comorbidities (n (%))				
Hypertension	40 (12.69)	33 (17.55)	7 (5.51)	0.0017
Diabetes mellitus*	28 (8.88)	18 (9.57)	10 (7.87)	0.0017
Dyslipidemia	34 (10.79)	27 (14.36)	7 (5.51)	0.6035
Stroke	4 (1.26)	3 (1.59)	1 (0.78)	0.5284
Gout	5 (1.58)	4 (2.12)	1 (0.78)	0.350
Hypothyroidism	6 (1.90)	4 (2.12)	2 (1.57)	0.726
Organ failure (n (%))**				
One organ	12 (3.80)	8 (4.25)	4 (3.14)	0.6139
>1 organ	8 (2.53)	5 (2.65)	3 (2.36)	0.8725
Other complication (n (%))				
Pneumonia	39 (12.38)	36 (19.14)	3 (2.36)	<0.0001
CRS	28 (8.88)	24 (12.76)	4 (3.14)	0.0033
Occupation-related exposure status (n (%))				
Direct exposure to patients	67 (21.26)	30 (15.96)	37 (29.13)	0.0052
Non-direct exposure to patients	248 (78.74)	158 (84.04)	90 (70.87)	0.0052

A total of 119 (37.77%) patients were found to be symptomatic with different symptom spectrums. Almost all symptomatic patients had flu-like symptoms (96.38%), and half of them had a cough (52.94%), while only one-third had shortness of breath (37.81%). Body ache and backache were reported by almost all symptomatic patients, while around 50% of the symptomatic patients had loose bowel motion or headache. Loss of smell or taste was found in a small percentage of patients (3.36%). The factors that may play a role in the association between hyperglycemia and COVID-19 infection are elaborated in Table 2.

**Table 2:** Characteristics of symptomatic COVID-19 patients according to their admission blood glucose level.

Parameters	Total (N=119)	Admission blood glucose < 140 mg/dL (n=73)	Admission blood glucose ≥ 140 mg/dL (n=46)	p-value
Age (years) (mean±SD)	43.31±13.66	36.00±12.39	50.76±11.46	<0.0001
Age (N (%))				
<40 years	52 (43.69)	43 (58.91)	9 (19.57)	<0.0001
>40 years	67 (56.31)	30 (41.09)	37 (80.43)	<0.0001
Gender (N (%))				
Male	73 (61.34)	44 (60.27)	29 (63.05)	0.9494
Female	46 (38.66)	29 (39.73)	17 (36.95)	0.7635
BMI (N (%))				
<30 (kg/m^2^)	78 (65.54)	57 (78.09)	21 (45.66)	0.0003
>30 (kg/m^2^)	41 (34.45)	16 (21.91)	25 (54.34)	0.0003
Comorbidities (N (%))				
Hypertension	39 (32.77)	14 (19.17)	25 (54.34)	0.0001
Diabetes mellitus*	27 (22.68)	7 (9.58)	20 (43.47)	0.0001
Dyslipidemia	33 (27.73)	10 (13.69)	23 (50)	<0.0001
Stroke	4 (3.36)	1 (1.36)	3 (6.52)	0.1285
Gout	5 (4.20)	2 (2.73)	3 (6.52)	0.3173
Hypothyroidism	6 (5.04)	2 (2.73)	4 (8.69)	0.1493
Other complications (N (%))				
Pneumonia	80 (67.22)	4 (5.47)	35 (70.06)	<0.0001
CRS	28 (23.52)	3 (4.10)	25 (54.34)	<0.0001
Disease severity (N (%))				
Mild	78 (65.56)	72 (98.64)	11 (23.98)	<0.0001
Severe	41 (34.44)	1 (1.34)	35 (76.02)	<0.0001
Fever (N (%))				
Low grade	67 (56.31)	60 (82.19)	6 (13.02)	<0.0001
High grade	52 (43.69)	13 (17.81)	40 (86.95)	<0.0001
Clinical presentation (N (%))				
Flu-like symptoms	115 (96.38)	71 (97.26)	44 (95.65)	0.341
Cough	63 (52.94)	22 (30.13)	41 (89.13)	<0.0001
Shortness of breath	45 (37.81)	8 (10.95)	37 (80.43)	<0.0001
Body ache	111 (93.27)	67 (91.78)	44 (95.65)	0.0864
Backpain	107 (89.91)	64 (87.67)	43 (93.47)	0.0887
Loss of bowel movement	46 (38.65)	23 (31.50)	23 (50)	0.0196
Loss of smell/taste	4 (3.36)	2 (2.73)	2 (4.34)	0.581
Headache	71 (59.66)	33 (45.20)	38 (82.60)	<0.0001
Clinical characteristics				
WBC count	7.56±3.56	6.26±2.01	7.35±3.64	0.035
Lymphocytes	1.13±0.45	2.10±0.85	1.10±0.39	<0.0001
Platelets	228.41±96.53	260.65±90.68	226.73±94.71	0.056
D-dimer (µg/mL)	1.17±1.09	0.40±0.36	1.14±1.01	<0.0001
ALT (U/L)	48 (27-100.25)	26.5 (23-45)	45.5 (28-89)	0.0268
LDH (U/L)	332 (282-427)	181 (141.5-232)	324.5 (242.75-401.7)	<0.0001
ALP (U/L)	80 (61-113)	77 (61-90)	81.5 (63.2-102.5)	0.037
BUN (mmol/L)	5.26±2.9	4.18±1.57	5.27±2.94	0.0095
Creatinine (µmol/L)	73.46±23.92	63.95±18.10	71.02±25.32	0.084
Ferritin (ng/mL)	1,536 (564-3,553)	166 (70.25-355)	979 (327.5-2,694.5)	0.001
CRP (mg/mL)	65 (36-90)	8 (5-24)	60 (38.2-89.2)	<0.0001

The mean age for the symptomatic patients was 43.1±13.66 years, out of which the majority were more than 40 years of age and had admission hyperglycemia (38.66%). The majority of symptomatic patients with hyperglycemia were males with BMI ≥ 30 kg/m^2^. Symptomatic patients with admission hyperglycemia had significantly higher rates of a history of hypertension, diabetes, and hyperlipidemia compared with normoglycemic patients. The rates of pneumonia or cytokine release syndrome were significantly higher in the hyperglycemia group. Additionally, patients with admission hyperglycemia experienced severe COVID-19 disease, were transferred to the intensive care unit, and received mechanical ventilation (1.3% versus 76.02% for patients without admission hyperglycemia versus those with admission hyperglycemia (p<0.0001)). Patients with admission hyperglycemia had higher chances of developing symptoms, especially cough, shortness of breath, loose bowel motion, and headache. Inflammatory markers were significantly higher among patients with hyperglycemia, especially WBC, D-dimer, ALT, LDH, and CRP, than those with normoglycemic levels.

The most significant clinical outcome related to COVID-19 can be classified into CRS and pneumonia. When looking at factors that are associated with those outcomes, age > 40 years and male gender were found to be significantly associated with the occurrence of the two conditions. Among comorbidities, hypertension was significantly associated with increased risk for the two conditions, diabetes was significantly associated with increased risk of CRS, and dyslipidemia was associated with a significantly increased risk of pneumonia. High-grade fever and shortness of breath were found to be significantly associated with increased risk of severity. Higher LDH and CRP levels were significant predictors for the two conditions (Table 3).

**Table 3:** Risk factors for severe COVID-19 outcomes using a multivariate regression model.

Risk factors	Cytokine release syndrome			Pneumonia		
	RR	95%CI	p-value	RR	95%CI	p-value
Age > 40 years	2.85	1.24-6.50	0.012	3.0	1.51-5.98	0.0007
BMI > 30 kg/m^2^	1.90	1.00-3.59	0.068	1.63	0.98-2.69	0.067
Male gender	2.95	1.35-6.41	0.0044	2.26	1.24-4.11	0.0058
Comorbidity						
Hypertension	2.73	1.43-5.20	0.003	1.97	1.20-3.23	0.011
Diabetes mellitus	2.32	1.25-4.32	0.018	1.51	0.86-2.56	0.165
Dyslipidemia	1.57	0.18-3.03	0.211	2.01	1.23-2.38	0.013
Asthma	2.26	0.94-5.40	0.141	1.56	0.67-3.65	0.329
Clinical presentation						
Flu-like symptoms (%)	-	-	-	-	-	-
Cough (%)	-	-	-	-	-	-
Shortness of breath (%)	21.37	5.32-85.80	<0.0001	30.42	7.70-120.1	<0.0001
Body ache (%)	-	-	-	-	-	-
Headache (%)	5.63	1.80-17.61	0.0005	4.59	1.93-10.91	<0.0001
High-grade fever (%)	36	5.05-256	<0.0001	24.66	6.23-97.63	<0.0001
Clinical investigations						
Admission hyperglycemia	13.22	4.20-41.32	<0.0001	13.88	5.28-36.50	<0.0001
Lymphocytes (<4)	-	-	-	-	-	-
LDH (>250 U/L)	12.32	3.94-38.54	<0.0001	8.13	3.69-17.90	<0.0001
Creatinine (>90 µmol/L)	2.14	1.09-4.21	0.056	1.40	0.75-2.63	0.391
Ferritin (>204 ng/mL)	-	-	-	-	-	-
CRP (>10 mg/mL)	16.41	2.30-116.71	<0.0001	11.25	2.84-44.44	<0.0001

Admission hyperglycemia was significantly associated with worse presentation and outcomes among symptomatic patients, where patients with admission hyperglycemia had almost three times increased risk of cough and seven times increased risk of shortness of breath (RR: 2.95 (2.05-4.25) and 7.33 (3.75-14.33), respectively). Additionally, admission hyperglycemia was associated with 13 times increased risk of pneumonia and CRS (RR: 13.88 (5.28-36.50) and 13.22 (4.2-41.32), respectively) (p<0.0001).

**Figure 1:**
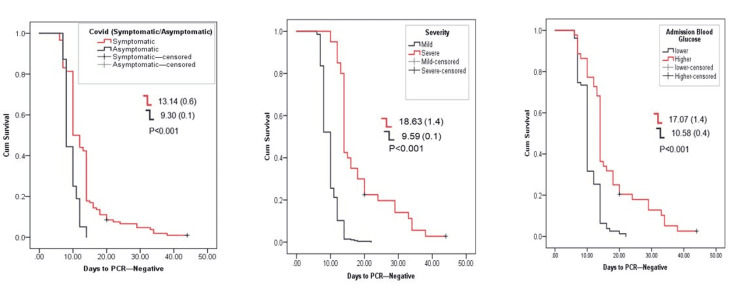
Association of clinical characteristics with days to negative PCR test.

**Figure 2:**
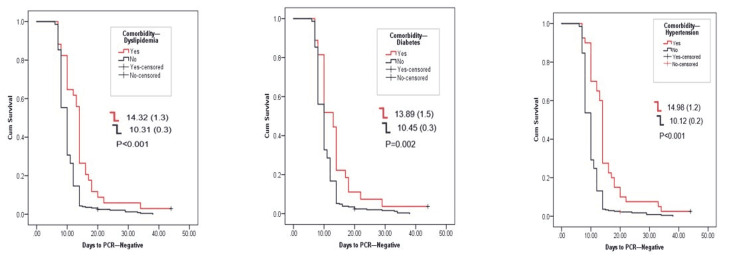
Association of different comorbidities with days to negative PCR test.

**Figure 3:**
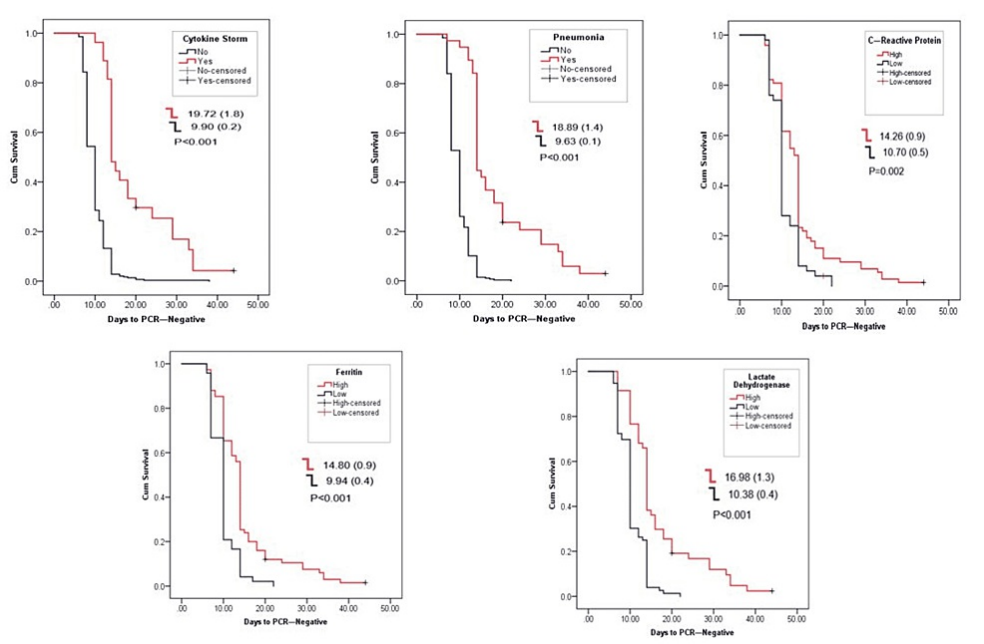
Association of different outcomes with days to negative PCR test.

All patients had negative PCR test before signing off their quarantine. Figure 1 shows the time length of negativization, showing the duration of being positive for the COVID-19 PCR test in relation to the clinical presentation, history of comorbidities, and biochemical markers, mainly CRP, ferritin, and LDH, and outcomes. In the studied cohort, asymptomatic patients became negative within 9.30±0.1 days, which is significantly earlier than the symptomatic patients with a mean of 13.14±0.6 days for negativization. This was also true for the severity and presence of hyperglycemia. The presence of comorbidities, namely, diabetes, dyslipidemia, and hypertension, had shown similar observation, where the presence of any of these three comorbidities will extend days to negativization from 13.89 to 14.98 days, while it was around 10 days for those who were not suffering from such comorbid condition (Figure 2). The occurrence of cytokine release syndrome and pneumonia extended the time to negativization to 19.72 and 18. 89 days, respectively, while elevated CRP, ferritin, and LDH all extended the time for negativization between 14 to more than 16 days (Figure 3).

Out of the total 315 patients, only two patients died. One patient was a 43-year-old female, and the other was a 58-year-old male with acute respiratory distress syndrome and cytokine storm as the leading causes of death. Both patients were obese with elevated LDH, ferritin, and admission hyperglycemia and lymphopenia. Only the male, but not the female, suffered from a history of diabetes, hypertension, and hyperlipidemia.

In the Appendices, the medications used to manage hospitalized symptomatic patients with COVID-19 infection are shown. A total of 63.9% were treated with antibiotics including meropenem (34.2%), cefepime (30.3%), azithromycin (22.4%), and ceftriaxone (13.1%). Antiviral medications (lopinavir/ritonavir) were prescribed for 37 (31.1%) symptomatic patients, while anticoagulants were prescribed for 46 (38.7%) patients. Hydroxychloroquine, tocilizumab, and methylprednisolone were prescribed at lower rates at 15.1%, 14.3%, and 12.6%, respectively.

## Discussion

This studied cohort was selected from Sultan Bin Abdulaziz Humanitarian City, Riyadh, Saudi Arabia, representing an isolated health sector community with 512 beds. This unique cohort represents both medical staff and general patients admitted for rehabilitation. The first case identified in this community was almost three months after the first case reported in the Kingdom among admitted patients. This indicates good adherence to all the preventive measures within the institution. The number of patients with confirmed COVID-19 infection increased with time, peaking from June 13 to 26, 2021, with 88-92 patients, especially among medical staff (Appendices).

The mean age for the studied cohort was 40 years, which is lower than what has been reported nationally [15] and internationally [16]. This is an expected finding since most of the studied population are employees in the Sultan Bin Abdulaziz Humanitarian City, who are mostly below 60 years of age. According to the findings of other studies [17], the most common comorbidities among our cohort were hypertension, hyperlipidemia, and diabetes mellitus. It was significantly higher among males, as was observed by a recent systematic review [18]. This could be explained by the fact that men are more exposed than women based on their cultural, social, and behavioral characteristics. On the other hand, women are generally able to mount a more vigorous immune response to infections and vaccinations [19]. Additionally, estrogen is associated with decreased expression of angiotensin-converting enzyme 2 (ACE2) receptors, which are the functional receptors for SARS-CoV-2 to enter the host target cell [20,21]. In men, testosterone is associated with suppressive effects on immune function, which may explain the greater susceptibility to infectious diseases observed in men [22]. In the current study, females were more significantly directly exposed to patients, which could be considered an occupational risk. It is well established that most frontline healthcare professionals are women. Further, women are more likely to serve as the primary caregivers within a household, thus becoming more exposed to the disease [23].

Hyperglycemia observed in this study was associated with older age, male gender, and a history of or prior comorbidities, i.e., diabetes, hypertension, and hyperlipidemia, especially among severe cases of COVID-19 infection. As has been observed in other studies, patients with higher admission blood glucose levels were older, predominantly male, and more frequently had a prior history of diabetes, hypertension, and other comorbidities [24]. This is also supported by the recent evidence that every 2 mmol/L (36 mg/dL) increase in fasting plasma glucose levels correlates with an increase in COVID-19 severity by 21% regardless of diabetes status [10]. This is once again clearly demonstrated in this study, where the mean blood sugar for severe cases was 189.57±60.41 mg/dL versus only 118.52±57.91 mg/dL for mild cases.

Factors associated with poor outcomes

Older age, male gender, hypertension, and diabetes were significantly associated with increased risk of CRS in the studied population. Such finding is well established in previous studies, where the male gender is one of the risk factors for increased severity and worse outcomes of COVD-19 infection [25,26]. This gender difference is probably due to biological and hormonal differences between the two genders, especially in the expression level of the ACE2 receptor to which SARS-CoV-2 binds [19-22]. Additionally, chronic comorbidities as clinical risk factors for a severe or fatal outcome associated with COVID-19 were elaborated on in a recent systematic review by Zhou et al. [27]. For instance, the results of a recent pooled analysis have shown that hypertension is associated with an up to 2.5-fold higher risk of severe and fatal COVID-19 [28], which is similar to our observation, where hypertension increased the risk for CRS by 2.73. Hypertension is a known clinical condition that predicts COVID-19 severity and may contribute to deterioration late in the disease course [29]. Since SARS-CoV-2 binds to ACE2 receptors to enter the cells, using angiotensin-converting enzyme inhibitors (ACEIs) and angiotensin receptor blockers (ARBs) as antihypertensive medications may be associated with enhanced ACE2 expression at the cell surface, thus eventually providing SARS-CoV-2 with a larger number of “anchors” for infecting cells [30,31]. Another explanation could be the fact that chronic comorbidities share several standard features with infectious disorders, such as a prolonged pro-inflammatory state and dysfunction of innate and adaptive immunity, which may be the key drivers of the worse clinical outcomes in patients infected with SARS-CoV-2 [32,33]. Although dyslipidemia was reported to potentially increase the mortality and severity of COVID-19 [34], it was associated with only an increased risk of pneumonia in the current study. BMI was not associated with any poor outcomes in this cohort as a result of the cohort being selected from medical personnel with a mean BMI of 25.4 kg/m^2^, especially when only 13% had BMI > 30 kg/m^2^.

Admission hyperglycemia as a risk factor for poor outcome

This is the same finding of a recent meta-analysis, where patients with admission hyperglycemia had an increased risk of severe/critical illness, implicating that hyperglycemia at admission may be an important predictive indicator of COVID-19 outcomes [35]. Although the underlying mechanisms of the impact of hyperglycemia at admission on COVID-19 outcomes are not fully investigated, there are several plausible explanations [35]. Patients suffering from severe COVID-19 have been observed to have “cytokine storms” or “inflammatory storms,” with higher levels of serum pro-inflammatory cytokines [36]. Of note, IL-6, a major pro-inflammatory cytokine, has been demonstrated to be significantly associated with the development or severity of COVID-19 in many studies [37,38]. Higher levels of IL-6 in patients with hyperglycemia or elevated blood sugar were observed on admission [39]. Therefore, hyperglycemia may induce a burst of IL-6 to participate in the progress of cytokine storm to aggravate COVID-19 symptoms.

Higher levels of LDH and CRP were also associated with an increased risk of poor outcomes among the studied cohort. This is in line with the studies that showed that CRP could serve as a candidate marker to predict the risk of worsening COVID-19 infection [40,41]. The elevated levels of CRP might be linked to the overproduction of inflammatory cytokines in patients with COVID‐19. In regard to LDH, its association with poor prognosis in patients with COVID-19 was reported in a recent meta-analysis [42]. Severe infections may cause cytokine-mediated tissue damage and LDH release, and since LDH is present in lung tissue (isozyme 3), it is expected that patients with the severe form of interstitial pneumonia will release greater amounts of LDH in the circulation [43].

Time length of negativization

Identifying subjects who are likely to have a long time to turn negative is crucial to prolong their isolation, once infected, and avoid the virus spread, especially in low- and middle-income countries, where RT-PCR tests are not freely available [44]. Thus, understanding factors associated with prolonged viral clearance is important to tailor prevention strategies. The minimum mean number of days for the studied cohort to convert to negative was 9.6 (±0.1) days, which is close to the finding of Ling et al. [45], which reported a median duration of viral shedding of 9.5 (6-11) days. The present study data indicate that symptomatic patients, patients with comorbid conditions (mainly diabetes and hypertension), patients with severe outcomes, and patients with elevated CRP, ferritin, and LDH tend to have a prolonged time of negativization, which was also observed in a Tunisian study [46] and Chinese study [47], where hypertension, diabetes, and disease severity delayed the SARS-CoV-2 virus clearance for extra five days for chronic complications and extra nine days for severe outcomes.

Limitations

This study has several limitations. It was a retrospective study, and some relevant data, such as smoking history, as well as some test results, such as IL-6, which is a known prognostic factor for COVID-19, were incomplete. Although the sample size was enough to assess the effect of hyperglycemia on COVID-19 severity, it was not enough to evaluate mortality. The main strength of this cohort is that it is a closed isolated community with a low incidence rate as a result of strict guidelines and limited contact with the community. Despite the limitations, the current study shed light on the fact that even in the absence of preexisting diabetes mellitus, hyperglycemia upon presentation is an independent risk factor for disease severity and poor COVID-19 infection outcomes. These patients must be identified and treated as such. To boost their outcomes, the COVID-19 vaccination program should also target those populations.

## Conclusions

In conclusion, the current study found that admission hyperglycemia was associated with an increased risk of progression to critical condition in patients hospitalized with COVID-19. Therefore, admission hyperglycemia should not be overlooked but rather should be detected and appropriately treated to improve the outcomes of COVID-19 patients with and without diabetes. Our findings suggested that patients with comorbidities, mainly hypertension, diabetes, and dyslipidemia, had greater disease severity than those without. Therefore, careful and structured past medical history collection should be implemented since this will help identify patients who would be more likely to develop serious adverse outcomes of COVID-19 and consequently need close monitoring. Post-COVID-19 isolation should depend on the case, where severe cases require almost double the time needed by mild cases.

## Figures and Tables

**Figure 4:**
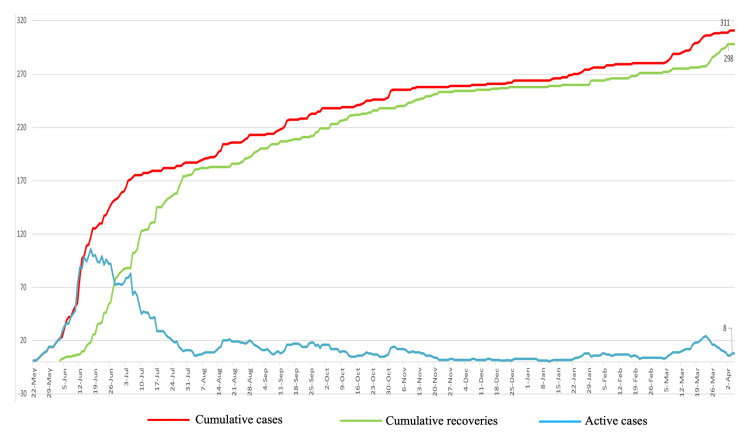
Dashboard for COVID-19 cases reported during the period from May 22, 2020, to April 2, 2021.

**Figure 5:**
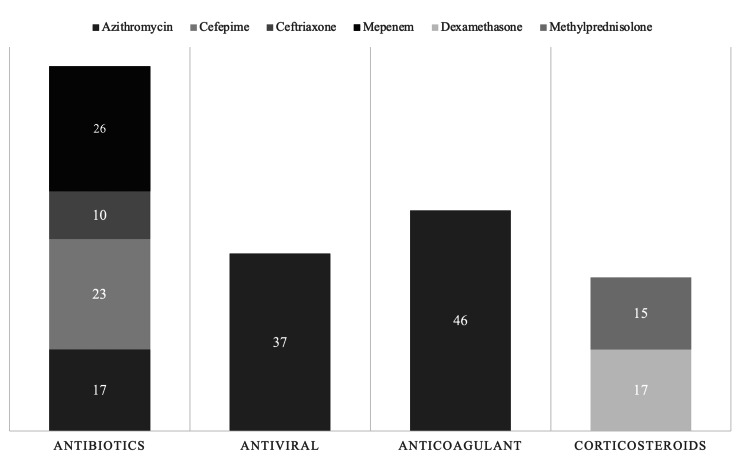
Management and outcome of all admitted COVID-19 patients (N=315).

## Appendices

The figures below show the dashboard for cases of COVID-19 reported from May 22, 2020, to April 2, 2021 (Figure 4) and the medications used to manage hospitalized symptomatic patients with COVID-19 (Figure 5).
